# Radiative and nonradiative relaxation phenomena in hydrogen- and oxygen-terminated porous silicon

**DOI:** 10.1186/1556-276X-9-47

**Published:** 2014-01-28

**Authors:** Neta Arad-Vosk, Amir Sa'ar

**Affiliations:** 1 Racah Institute of Physics and the Harvey M. Kruger Family Center for Nanoscience and Nanotechnology, The Hebrew University of Jerusalem, Jerusalem 91904, Israel

**Keywords:** Porous silicon, Photoluminescence, Quantum confinement, Surface chemistry, Nonradiative processes, The vibron model

## Abstract

**Abstract:**

Using time-resolved photoluminescence spectroscopy over a wide range of temperatures, we were able to probe both radiative and nonradiative relaxation processes in luminescent porous silicon. By comparing the photoluminescence decay times from freshly prepared and oxidized porous silicon, we show that radiative processes should be linked with quantum confinement in small Si nanocrystallites and are not affected by oxidation. In contrast, nonradiative relaxation processes are associated with the state of oxidation where slower relaxation times characterize hydrogen-terminated porous silicon. These results are in a good agreement with the extended vibron model for small Si nanocrystallites.

**PACS:**

78.55.Mb; 78.67.Rb; 78.47.jd

## Background

The efficient room-temperature visible photoluminescence (PL) from porous silicon (PSi) has attracted much attention in recent years, mainly due to open questions and controversies concerning the mechanism responsible for the PL emission [1-7]. In addition, numerous PSi-based devices having potential applications in diverse fields such as photonics, optoelectronics, and photovoltaics, were proposed and investigated [8-15]. In particular, PSi has been considered as an attractive candidate for sensing applications [16-21] where its large surface area can be exploited for enhancing the sensitivity to surface interactions. In such a sensor, the PL emitted from PSi can be used as a transducer that converts the chemical interaction into a measurable optical signal. For example, PL quenching due to surface interactions with various chemical species has been utilized for developing various biophotonic sensors [16,22,23].

Originally, the efficient PL from PSi was attributed to quantum confinement (QC) of charged carriers in Si nanocrystallites located in the PSi matrix [24]. Experimental evidences supporting this model include a shift of the energy bandgap with size [1-3,25,26], resonant PL at low temperatures [27-29], and PL decay time spectroscopy [1,2,27]. However, the QC model cannot account for other experimental observations, mainly the dependence of the PL on surface treatments [30-34]. Several reports proposed a more complex picture of QC combined with localization of charged carriers at the surface of the nanocrystals [35-38], particularly the work of Wolkin et al. [36] who demonstrated a strong dependence of the PL on surface chemistry. This group has shown that while in fresh PSi the PL peak energy depends on the size of the nanocrystals (i.e*.*, follows the QC model), the QC model cannot account for the limited PL shift observed for oxidized PSi. By introducing surface traps into the model, the behavior of the PL peak energy for oxidized PSi could be explained [36]. Other reports have shown that both QC and surface chemistry shape the PL characteristics [37,38]. The extended vibron (EV) model provides a simple explanation to the mutual role of surface chemistry and QC [39-41]. According to this model, QC affects radiative processes that are less sensitive to the state of the surface, while nonradiative relaxation processes are mostly influenced by the surface chemistry. However, both QC and surface chemistry contribute to the efficient PL from PSi.

In this work, we investigate the role of surface chemistry, particularly the relationship between the state of oxidation and the PL characteristics of luminescent PSi samples. We examine the contribution of radiative and nonradiative decay processes to the overall PL lifetime and the sensitivity of these processes to surface treatments. Furthermore, we examine the EV model by comparing radiative and nonradiative decay times of freshly prepared hydrogen-terminated PSi (H–PSi), with those of oxidized PSi (O–PSi). This allows to experimentally test the hypothesis that radiative processes are not sensitive to surface treatments while nonradiative processes are. Utilizing temperature-dependent, time-resolved PL (TR-PL) spectroscopy [42], we extend our previous work on silicon nanocrystals embedded in SiO_2_ matrices and silicon nanowires [37,41,43,44] to PSi, as this system allows a modification of the surface chemistry by simple means and tracing quite accurately the state of the surface.

## Methods

PSi samples were prepared by electrochemical etching of p-type (10 to 30 Ω⋅cm) silicon wafers under standard dark anodization conditions [25,26]. A 1:1 mixed solution of aqueous hydrofluoric (HF) acid (49%) and ethanol was used as the electrolyte at a current density of 70 mA cm^-2^ for 200 s to yield a PSi layer of approximately 9.5 μm (measured by scanning electron microscope) with average pore size of a few nanometers [25]. The freshly prepared PSi is terminated by Si-hydrogen bonds that are known to be quite unstable under ambient conditions. These bonds are subsequently replaced by the more stable Si-oxygen bonds upon exposure to air. Hence, in order to investigate the optical properties of H–PSi, we introduced the freshly prepared samples into a vacuum optical cryostat and kept them under vacuum conditions for the entire experiment. Oxygen-terminated O–PSi was obtained after taking the same PSi sample out of the vacuum cryostat and letting it age under ambient conditions for 6 days. The state of the PSi surface (having either Si-O or Si-H bonds) was monitored by Fourier transform infrared (FTIR) spectroscopy. To eliminate interference phenomena, thinner PSi samples were prepared for these measurements (10 s of anodization under the same conditions, resulting in approximately 450 nm thick PSi film). Bruker's Vertex-V70 vacuum FTIR spectrometer (Bruker Optik GmbH, Ettlingen, Germany), equipped with a mercury-cadmium-telluride (MCT) photovoltaic detector, has been exploited for these experiments. Measurements were performed in the grazing angle reflection mode, at an incidence angle of 65° and under p-polarization (to enhance the sensitivity to surface bonds [45]). For continuous wave (cw) PL and TR-PL measurements, the samples were excited by Ar^+^ ion laser operating at 488 nm while the PL signal was dispersed by a 1/4-m monochromator and detected by a photomultiplier tube. For time-resolved measurements, the laser beam was modulated by an acousto-optical modulator driven by a fast pulse generator, while the PL signal has been analyzed by a gated photon counting system. During PL measurements, the samples were kept under vacuum, in a continuous-flow liquid helium optical cryostat that allows temperature control from approximately 6 K up to room temperature.

## Results

**Figure 1:**
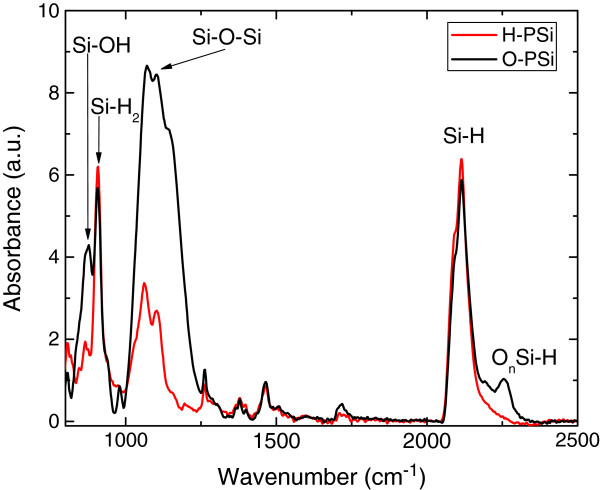
**FTIR spectra.** Infrared absorption spectra of H-PSi (freshly prepared PSi) and O-PSi (the same sample after aging). Main Si-H, Si-OH, and Si-O vibration modes are marked.

IR absorbance spectra of H–PSi (red line) and O–PSi (black line) are presented in Figure 1. Si-OH and Si-O-Si vibrational bands at 875 cm^-1^ and 1,065 to 1,150 cm^-1^ respectively [46-48], which indicate the presence of oxygen in the films, clearly increase after 6 days of exposure to ambient atmosphere. The Si-hydrogen vibrations [46,47] at 906 cm^-1^ and 2,112 cm^-1^ did not fade away after 6 days of exposure, and typically disappear only after several weeks of exposure to air [49]. Hence, these IR absorbance spectra confirm the modification of the PSi's surface during the exposure to air.

**Figure 2:**
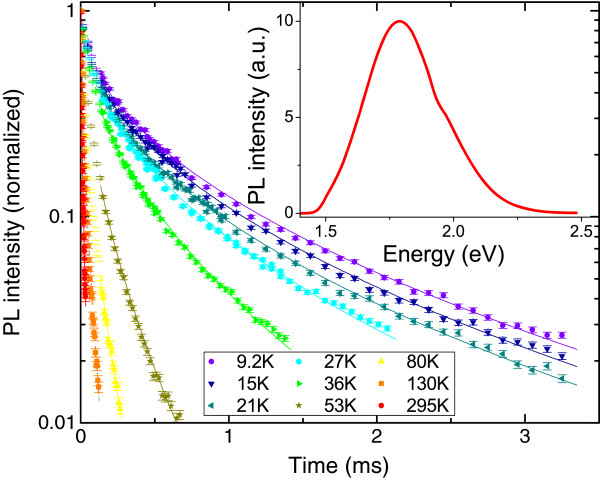
**PL decay curves.** The PL decay curves of H-PSi measured at a photon energy of 2.03 eV (610 nm) and at various temperatures. The solid lines present the best fit to a stretched exponential function (Equation 1). Inset shows the PL spectrum of H-PSi measured at room temperature.

**Figure 3:**
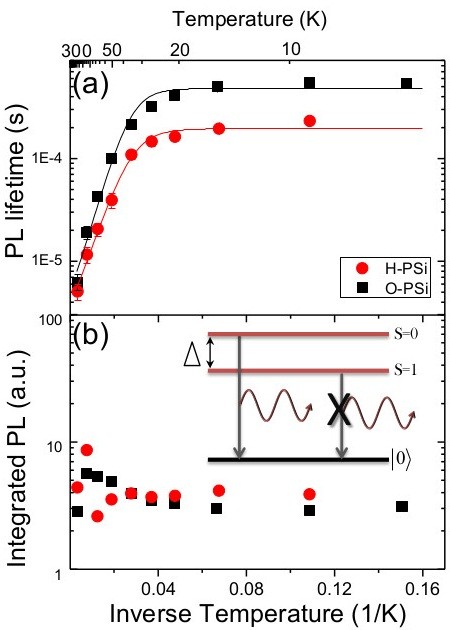
**PL lifetime and integrated PL. (a)** Arrhenius plot of the PL lifetime (on a semi-logarithmic scale) as a function of 1/*T*, at a photon energy of 2.03 eV (610 nm) for H-PSi (red circles) and O-PSi (black squares). The solid lines represent the best fit to the singlet-triplet model of Eq.2. **(b)** Arrhenius plot of the integrated PL. Inset shows the schematics of the excitonic two-level model with the upper excitonic singlet-triplet state and the ground (no exciton) state. The arrows represent the allowed (from the singlet) and the forbidden (from the triplet) optical transitions.

The cw-PL spectrum of H-PSi, measured at room temperature with a PL maximum at approximately 1.80 eV (about 690 nm) and a full width at half maximum (FWHM) of about 0.4 eV, is presented at the inset to Figure 2. A similar spectrum with a slight blue shift of the PL maximum to 1.85 eV (approximately 670 nm) has been measured for O-PSi, in agreement with results obtained in references [50-52]. In order to probe both radiative and nonradiative relaxation processes, the PL decay curves were measured at several photon energies and at temperatures ranging from 6 K up to room temperature. As will be discussed and explained later on, at room temperature radiative processes dominate over nonradiative processes and therefore, for the study of nonradiative processes, it is necessary to measure the PL decay at low temperatures. Typical PL decay curves, measured for H-PSi at a photon energy of 2.03 eV (610 nm) and at various temperatures, are presented in Figure 2. A pronounced dependence of the PL decay on temperature can clearly be seen, similar to the results of other groups [1,2,53]. As the temperature decreases, the PL decay time becomes significantly longer (by two orders of magnitude over the entire range of measured temperatures). Notice that the temporal behavior of the PL cannot be described by a simple exponential decay function (see the semi-logarithmic scale of Figure 2) and is typically fitted to a stretched exponential decay function [54,55]. This nonexponential decay is common to disordered systems and has been attributed to a dispersive diffusion of the photo-excited carriers [54]. The solid lines in Figure 2 represent the best fit of the PL decay curves to a stretched exponential function, given by

(1) $$I\left(t\right)=I_{0}\exp\left[-{\left(t/\tau \right)}^{\beta }\right]$$

where *τ* is the PL lifetime, and *β* is the dispersion exponent that was found to vary in between 0.4 to 0.8 and will not be discussed here (see [37] for more details). Arrhenius plot (semi-logarithmic scale versus the inverse temperature) of the measured PL lifetime for both H- and O-PSi (at a photon energy of 2.03 eV) is shown in Figure 3a, presenting exponentially fast decays at high temperatures and approximately long and constant decay times at low temperatures. This unique behavior of the PL decay has been attributed to a splitting of the excitonic ground state (i.e., the photo-excited electron–hole pair) due to the Coulomb exchange interaction, giving rise to a lower triplet level (*S* = 1) and an upper singlet level (*S* = 0) [53] (see inset to Figure 3b). The splitting is further enhanced by confinement of the charged carries in small nanocrystals, giving rise to a larger excitonic overlap. Optical transitions from the lower triplet and the upper singlet states are forbidden and allowed respectively, due to spin selection rules [1,2,39,40,53]. However, the lifetime of the triplet state becomes weakly allowed due to spin-orbit interaction [39,40,53]. Hence, the triplet lifetime is expected to be considerably longer than the singlet lifetime. At low temperatures (where *kT* < < Δ, and Δ is the singlet-triplet splitting energy; see inset to Figure 3b), only the triplet level is populated and therefore, the PL decay time is dominated by the triplet lifetime and is relatively long (the low-temperature plateau regions in Figure 3a). As the temperature increases (above approximately 30 K), the upper singlet level becomes thermally populated and the overall lifetime shortens according to the following expression:

(2) $$\frac{1}{\tau_{R}}=\frac{\frac{g}{\tau_{L}}+\frac{1}{\tau_{U}}\exp\left(-\Delta /\mathrm{kT}\right)}{g+\exp\left(-\Delta /\mathrm{kT}\right)}$$

where *τ*_R_ stands for the radiative decay time and *τ*_L_ and *τ*_U_ are the lower triplet and the upper singlet lifetimes respectively (*g* = 3 is the levels degeneracy ratio) [1,39,40,53]. At high temperatures, the decay time is dominated by the much faster upper singlet lifetime.

**Figure 4:**
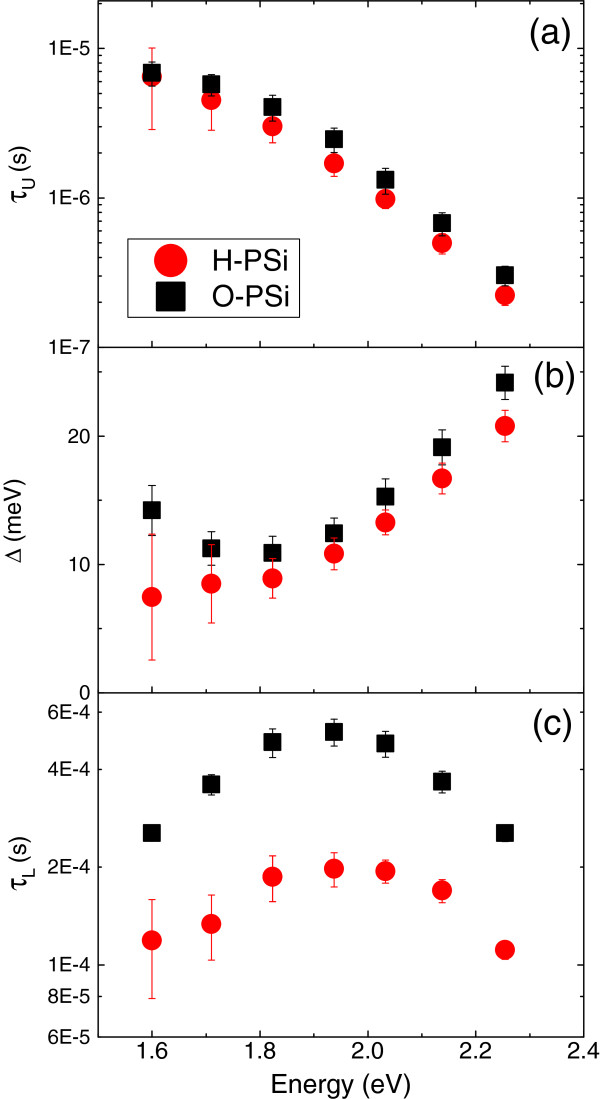
**Triplet and singlet lifetimes and energy splitting. (a)** the upper singlet lifetime; **(b)** the excitonic energy splitting; **(c)** the lower triplet lifetime (extracted from the fit to the singlet-triplet model; see Figure 3) as a function of the photon energy.

From Figure 3a we found that within the experimental errors, the upper singlet decay times of H- and O- PSi (at photon energy of 2.03 eV) are essentially the same (1.0 ± 0.2 μs and 1.3 ± 0.2 μs for H-PSi and O-PSi, respectively). However, at low temperatures the H-PSi decay time is faster than that of the O-PSi (200 ± 50 μs relative to 480 ± 50 μs, respectively). To further explore the differences between H- and O- PSi decay times, the singlet and the triplet lifetimes as well as the energy splitting were extracted over the measurement's range of photon energies and are plotted in Figure 4. As expected, the upper singlet lifetime (*τ*_U_) is significantly shorter (by about one to two orders of magnitude) than the lower triplet lifetime (*τ*_L_) over this range of photon energies. The energy splitting, Δ*E* (see Figure 4b), was found to vary between 7 and 25 meV, as in [53] and in accordance with the calculated values in [56-58]. Comparing H- and O- PSi, we note that the upper singlet lifetimes and the excitonic energy splitting of both H-PSi and O-PSi remarkably coincide over the entire range of measured photon energies (see Figure 4a,b), while the lower triplet lifetime of H-PSi is shorter than that of O-PSi over the same range of energies (Figure 4c). This result is the basis for our conclusion (to be discussed hereafter) that oxidation of (freshly prepared) H-PSi gives rise to slower nonradiative lifetimes, leaving radiative lifetimes unaffected.

## Discussion

As explained above, the main finding of this work is that the oxidation of freshly prepared luminescent PSi gives rise to slower triplet lifetimes, keeping the upper singlet lifetimes unaffected. Before discussing the implications of this result, let us denote that the measured decay rate is the sum of two competing relaxation processes given by

(3) $$\tau^{-1}=\tau_{R}^{-1}+\tau_{\mathrm{NR}}^{-1}$$

where *τ*_R_^-1^ is the radiative transition rate (given by Equation 2), *τ*_NR_^-1^ is the nonradiative relaxation rate, and *τ*^-1^ is the total decay rate. The integrated PL (i.e*.*, the area below the PL spectrum shown at the inset to Figure 1) is proportional to the quantum yield that is given by the ratio of the radiative to the total decay rate, $\eta =\tau_{R}^{-1}/\tau^{-1}=\tau /\tau_{R}$. The variation of the integrated PL with temperature is shown in Figure 3b on a semi-logarithmic scale, similar to that of Figure 3a for the PL lifetime. Notice that while the PL lifetime varies by approximately two orders of magnitude over the 30 to 300 K temperature range, the integrated PL varies by less than 3. Hence, one concludes that at this temperature range, *τ*_R_ < < *τ*_NR_, leading to, *τ* ≈ *τ*_R_ (Equation 3), and *η* ≈ constant (as in reference [37]). Thus, at temperatures above 30 to 40 K the measured lifetime is dominated by radiative transitions. In addition, the strong dependence of the upper singlet lifetime on photon energy (a decrease from 6 to 7 μs at 1.6 eV down to 200 to 300 ns at 2.3 eV; see Figure 4a), suggests again that this lifetime should be associated with radiative transitions (where *τ*^U^ ~ *τ*_R_^U^ < < *τ*_NR_^U^). In this case, the fast radiative lifetime is due to the influence of confinement on the spontaneous emission rates in small Si nanocrystals [39,40]. On the other hand, the lower triplet lifetime that is dominant at low temperatures is approximately constant (varies by less than factor of 2 over the same range of energies) and roughly independent of the photon energy that probes a given size of nanocrystals. This suggests that the low-temperature lifetime should be associated with a nonradiative relaxation time (of the whole system) that dominates over the (forbidden) radiative triplet lifetime, in agreement with [37,39,40].

Turning back to our main findings, we conclude that oxidation (in ambient conditions) has a minor impact on the size of the nanocrystals (giving rise to about 3% blue shift of the PL spectrum) and no noticeable effect on the radiative lifetime and the excitonic energy splitting (via their dependence on photon energy). On the other hand, nonradiative relaxation times, which are associated with the state of the surface, are expected to be sensitive to oxidation and to a modification of surface bonds as experimentally observed (see Figure 4c). This result can be explained by the EV model [39,40], which assigns the slow nonradiative relaxation times to resonant coupling between surface vibrations and quantized electronic sublevels in the conductance/valence bands of the nanocrystals. The stronger is the coupling between these electronic states and surface vibrations, the slower are the nonradiative lifetimes [39,40]. Hence, according to this model, the longer lifetime measured for O-PSi (compared to H-PSi) should be assigned with the larger electronegativity of oxygen (relative to hydrogen) that gives rise to larger dipole strength of the Si-O-Si vibration [47].

Finally, let us point out that the conclusion *τ*_R_ *< < τ*_NR_ (for both types of PSi; see Figure 4) implies that the PL quantum yield should approximately be constant. This conclusion provides a simple explanation to the slight variation of the PL intensity under oxidation, as oxidation modifies nonradiative relaxation times associated with the PSi surface. However, this has a minor impact on the PL quantum yield as the PL efficiency is practically independent of the nonradiative relaxation times at high temperatures [39,56,57] and is mostly influenced by the nanocrystals size [59,60].

## Conclusions

In conclusion, using temperature-dependent, time-resolved PL spectroscopy for probing both radiative and nonradiative relaxation processes in freshly prepared and oxidized PSi, we were able to show that radiative processes should be associated with quantum confinement in the core of the Si nanocrystallites and therefore, are not affected by oxidation. On the other hand, nonradiative relaxation processes are affected by oxidation and by the state of the nanocrystallites surface. These results are consistent with the extended vibron model that assigns radiative relaxation to QC, while nonradiative processes are assigned to surface chemistry.

## Abbreviations

EV: Extended vibron; H–PSi: Hydrogen-terminated porous silicon; O-PSi: Oxidized porous silicon; PL: Photoluminescence; PSi: Porous silicon; QC: Quantum confinement; TR-PL spectroscopy: Time-resolved photoluminescence spectroscopy.

## Competing interests

The authors declare that they have no competing interests.

## Authors’ contributions

NAV carried out the experiments, contributed to the interpretation of the data and drafted the manuscript. AS contributed to the interpretation of the data and revision of the manuscript. Both authors read and approved the final manuscript.
